# SlPIN1 regulates auxin efflux to affect flower abscission process

**DOI:** 10.1038/s41598-017-15072-7

**Published:** 2017-11-02

**Authors:** Zihang Shi, Yun Jiang, Xinqi Han, Xin Liu, Ruishu Cao, Mingfang Qi, Tao Xu, Tianlai Li

**Affiliations:** 1 College of Horticulture, Shenyang Agricultural University, Shenyang, 110866 Liaoning P. R. China; 2 Key Laboratory of Protected Horticulture of Ministry of Education, No. 120 Dongling Road, Shenhe District, 110866 P. R. China

## Abstract

*Solanum lycopersicum* PIN-FORMED1 (SlPIN1), a major auxin efflux facilitator, contributes to the establishment of auxin maxima during organ initiation and development in tomato. However, the functions of SlPIN1 during organ abscission remain unclear. In our study, *SlPIN1* expression decreased immediately after flower removal and increased following IAA treatment, indicating a high sensitivity to auxin depletion. 1-MCP (an ethylene inhibitor) delayed abscission and down-regulated *SlPIN1*, indicating that ethylene may positively regulate *SlPIN1* and that low expression levels of *SlPIN1* may delay abscission. The SlPIN1 protein levels were not consistent with the expression pattern, implying that in addition to transcription, protein degradation also affects SlPIN1 levels during abscission. The phosphorylation of SlPIN1 at Ser418, which significantly declined during abscission, was found to play roles in SlPIN1 localization and auxin transport. We also identified the interaction proteins of SlPIN1, which were involved in phosphorylation and ubiquitylation. Therefore, complex mechanisms mediate SlPIN1 auxin transport capability during abscission. The silencing of *SlPIN1* expression accelerated abscission by increasing auxin accumulation in the ovary and decreasing the auxin content in the abscission zone (AZ), indicating that SlPIN1 plays a major role in mediating auxin source-sink transport and the establishment and maintenance of auxin maxima in the AZ.

## Introduction

Polar auxin transport (PAT) controls developmental events, such as root formation^1,2^, leaf morphogenesis^3^, vascular formation and patterning^4–6^, fruit development^7,8^ and seed dispersal^9^, by creating auxin maxima and minima. Organ abscission is a highly sequential process that results from the coordinated regulation of auxin and ethylene. Auxin is normally transported from source tissues, such as young leaves, flowers and developing seeds, to the plant by basipetal PAT^7,10^. The blocking of normal basipetal PAT in the abscission zone (AZ) sensitizes the AZ to ethylene, which is responsible for abscission^11–13^. Compared with other auxin maxima-dependent processes, the decline in auxin levels in the AZ is critical for the timing of abscission. Reducing the indole-3-acetic acid (IAA) levels in the AZ specifically accelerated abscission in *Arabidopsis*, while enhancing the IAA levels delayed abscission^14^. A close relationship between a decrease of in the IAA concentration in the AZ and the triggering of pedicel abscission was also observed in tomato^15^. The decline of auxin in the AZ leads to modified expression of auxin-regulated genes, which sensitizes the AZ to ethylene^11^. Ethylene in turn leads to the activation of genes related to ethylene signal transduction, cell wall degradation and programmed cell death, and pathogenesis-related defence and defence layer development^11,13,16,17^, which are involved in abscission execution and defence layer differentiation. However, although the auxin levels in the AZ continue to decrease during abscission, the molecular mechanisms underlying PAT regulation through the pedicel remain unclear.

Unlike the apoplast, the weak alkaline environment in the cytosol prevents auxin from passing through the plasma membrane (PM) in its deprotonated form. Auxin efflux facilitators, i.e., PIN proteins^18^ and ABCB proteins^19^, enable the intercellular transport of auxin. Eight PIN proteins have been identified in *Arabidopsis*, and most PIN proteins are localized asymmetrically at the PM^4,18,20,21^. Due to their asymmetric localization, the PIN proteins play a very important role in the determination of the PAT direction and the establishment of auxin gradients in tissues^10,22–25^.

The *PIN* genes are regulated by hormones. Auxin regulates the expression of *PIN* via an Aux/IAA-ARF-dependent feedback mechanism^26–29^, and putative auxin responsive elements (AuxREs) have been found in all eight *AtPIN* promoter sequences^30^. The complex consisting of ARF5 and IAA12 has been reported to have an influence on *PIN1* gene expression and the polar localization of the PIN1 protein in the PM^31–33^, although the mechanism is unclear. In addition, the sensitivities to auxin vary depending on the *PIN* genes and tissues^30^. Ethylene also plays roles in the regulation of *PIN* expression, particularly for PM-localized PINs. Ethylene has been shown to enhance PAT in roots by up-regulating the expression levels of *PIN1*, *PIN2* and *PIN4*, resulting in an increased auxin distribution in the root and the inhibition of root elongation^34^. Furthermore, ethylene enhances root IAA transport by increasing the expression of *PIN3* and *PIN7*, which inhibits lateral root development^35^. While the roles played by ethylene in the abscission process have been reported^11,13,16,17^, the influence of ethylene on pedicel auxin transport and *PIN* expression remains unknown.

PM-localized PINs do not remain in the PM; trafficking between the trans-Golgi network and the PM, which is called PIN recycling, is normal for PM-localized PINs. This recycling is essential for PIN auxin loading^36^ and polar localization on the PM^37,38^. PIN recycling is a complex and highly controlled process that is involved in many posttranslational modifications of PINs, such as phosphorylation^39–41^, ubiquitylation and degradation^42,43^. The functions of phosphorylation are related to the polar localization of PINs. The kinases PINOID (PID)^39^, WAG1, WAG2^44^, and D6 PROTEIN KINASES (D6PKs)^41^, and one phosphatase PROTEIN PHOSPHATASE 2 A (PP2A)^40^, are involved in PIN phosphorylation and dephosphorylation. Overexpression of PID or phosphomimetic substitutions at the PID phosphorylated sites of PIN1 result in a basal-to-apical shift of PIN1 localization in *Arabidopsis* ^39,45^, while dephosphorylation induced by PP2A has an antagonistic function^40^. Furthermore, other kinases, such as the D6PKs, affect the auxin transport activity of PIN rather than its localization^41^. Although some phosphorylation sites are regulated by PID, many phosphorylation sites are phosphorylated by unknown kinases^30^, indicating the complexity and diversity of PIN phosphorylation. Ubiquitylation plays a regulatory role during PIN recycling. Ubiquitylation is involved in PIN2 turnover and the asymmetric distribution of auxin during the gravitropic response^42^. However, knowledge regarding PIN ubiquitylation in other PINs or other physiological processes is limited.

AtPIN1 was the first identified and is the most important member of the PIN family in *Arabidopsis*, and only the *pin1* mutant among the *PIN* single mutants exhibits severe developmental defects, such as an abnormal phyllotactic patterning and lateral organ initiation inability^4,46,47^. SlPIN1, which is an orthologue of AtPIN1 in tomato, also plays central roles in leaf initiation and fruit development. In the tomato leaf primordia, SlPIN1 localizes in the leaflet and lobe initiation site and generates auxin maxima for the initiation of leaflets and lobes^48^. In addition, SlPIN1 is highly expressed in the placenta, particularly during the early stages of fruit development, suggesting that SlPIN1 plays a role in auxin transport from the seed to the plant^7^.

Although the role of auxin depletion in abscission triggering has been largely described, information regarding auxin transport and its molecular mechanisms during the abscission process is limited. Hence, in this study, we attempted to reveal the possible roles of SlPIN1 in dynamic auxin transport and pedicel abscission. We characterized the temporal and spatial expression of *SlPIN1* in the tomato pedicel during abscission. Changes in the SlPIN1 protein in the AZ and its phosphorylation and interaction proteins were also investigated, and the possible roles of SlPIN1 in basipetal auxin transport and auxin decrease in the AZ were revealed. A virus-induced gene silencing (VIGS) transgenic line of *SlPIN1* was generated to provide additional evidence regarding the role of this gene in the regulation of tomato pedicel abscission.

## Results

### Expression of *SlPIN1* exhibits a temporal- and spatial-specific pattern during tomato flower abscission

**Figure 1:**
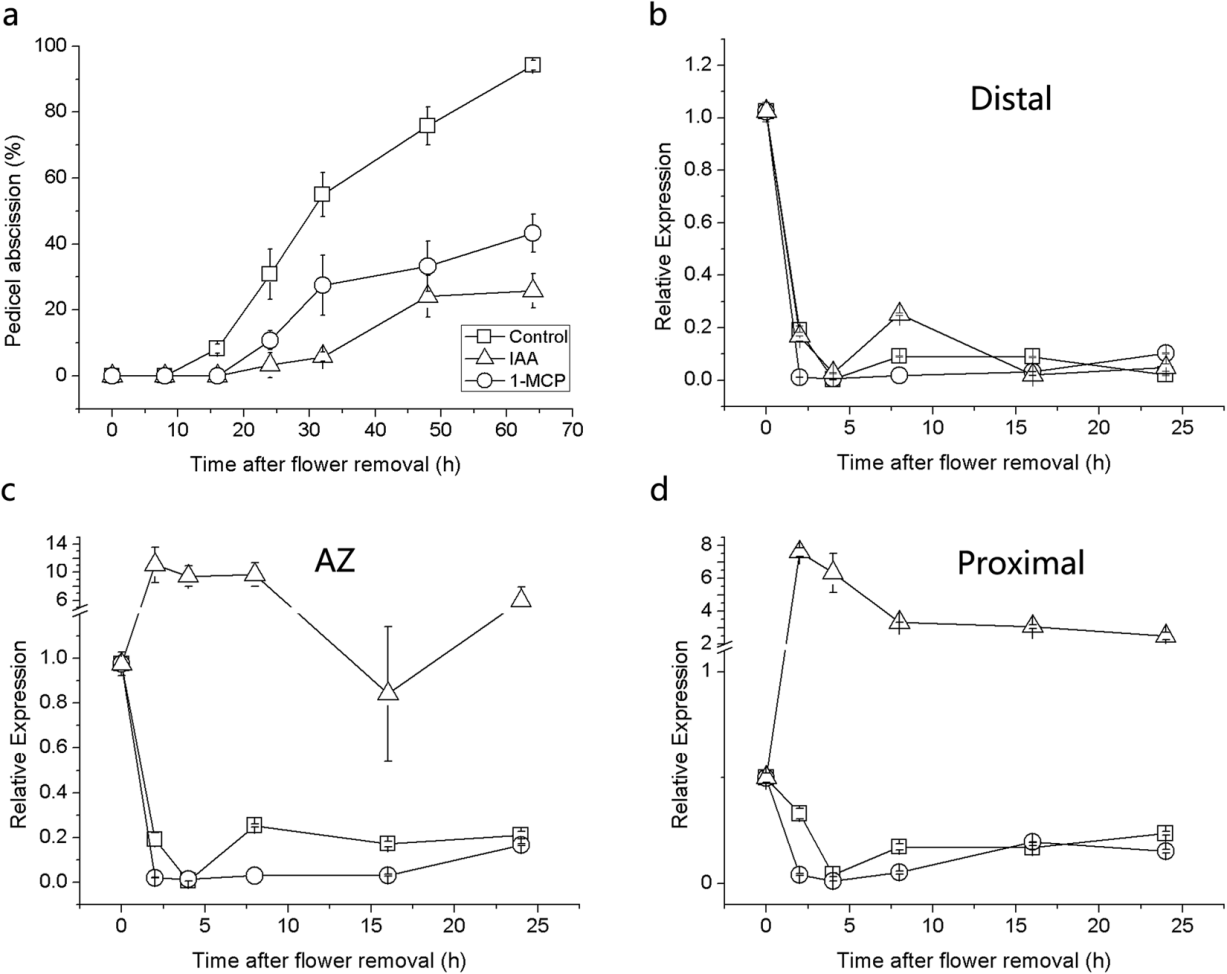
Abscission rates and *SlPIN1* expression profiles in the pedicel during abscission following different treatments. The pedicels were incubated in agar, 50 μg/g IAA, or 20 μL/L 1-MCP. (**a**) IAA and 1-MCP delayed abscission. The data are presented as the mean (±SD) values corresponding to three independent tests, and for each test, 40 to 50 pedicels were investigated. (**b**–**d**) *SlPIN1* expression in the distal part (B), the AZ (C) and the proximal part (D) during abscission. The data are presented as the mean (±SD) values corresponding to three independent tests.

Normally, the tomato pedicel begins to abscise 16–24 h after flower removal, and a treatment (at the proximal end of the pedicel explants) with IAA and 1-methylcyclopropene (1-MCP, inhibitor of ethylene binding to receptor) delayed the abscission process (Fig. 1a). To characterize the expression pattern of *SlPIN1* in the tomato pedicel during abscission, flower pedicel explants were sampled 0–24 h after flower removal, and *SlPIN1* expression in the distal part, the AZ and the proximal part were analysed by quantitative real-time PCR (RT-PCR). Before the flower removal, the *SlPIN1* expression in the proximal part was lower than that in the distal part or the AZ, and all three parts showed down-regulated expression levels within 4 h after the flower removal. After 4 h, *SlPIN1* was up-regulated in the AZ and proximal part, while its expression remained low in the distal part for 24 h (Fig. 1b,c,d, Control).

To further investigate the relationship between *SlPIN1* and the abscission process and the *SlPIN1* expression in response to auxin and ethylene, the two major hormones in abscission were analysed using IAA and 1-MCP treatments. IAA prominently increased the *SlPIN1* expression within 24 h in the AZ and proximal part but not in the distal part (Fig. 1b,c,d, IAA). Because the pedicels were treated at the proximal end, the IAA uptake in the distal part may have been influenced by the direction of PAT; thus, we treated the distal end with IAA, and re-examined the *SlPIN1* expression. The distal part showed a similar up-regulation of *SlPIN1* expression following the IAA treatment (Supplementary Fig. S1b), revealing a consistent *SlPIN1* response to IAA in the pedicel. In addition, the IAA treatment at the distal end completely inhibited pedicel abscission, and no abscised pedicels were observed within 64 h after the flower removal (Supplementary Fig. S1a). The ethylene action inhibitor 1-MCP depressed the *SlPIN1* expression at an earlier stage (0–8 h) (Fig. 1b,c,d).

**Figure 2:**
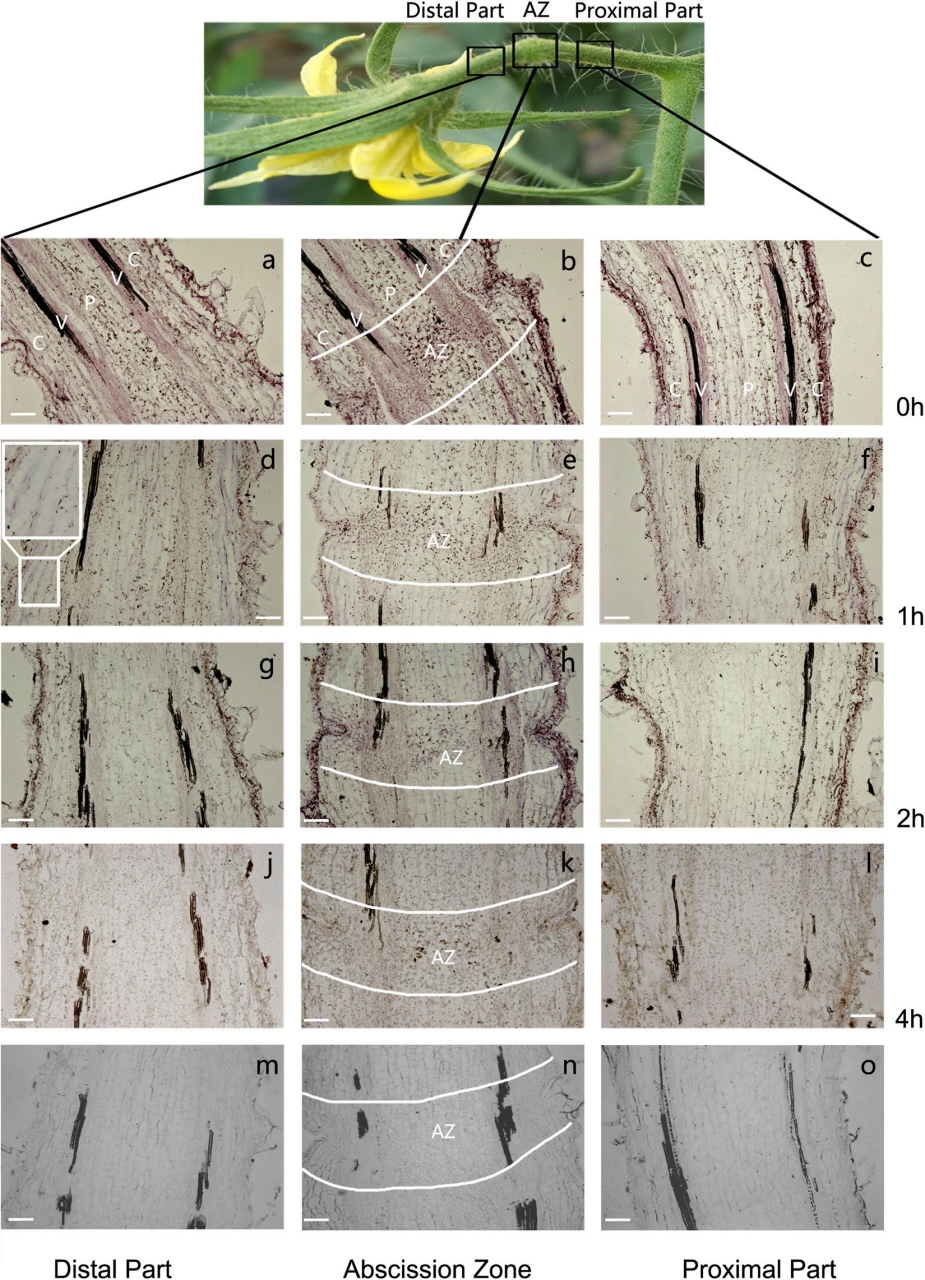
*In situ* hybridization of *SlPIN1* in the tomato pedicel during abscission. Images of longitudinal sections of the distal part (**a**,**d**,**g**,**j**), AZ (**b**,**e**,**h**,**k**) and proximal part (**c**,**f**,**i**,**l**) 0 h (a-c), 1 h (**d**–**f**), 2 h (**g**–**i**) and 4 h (**j**–**l**) after flower removal and detection of *SIPIN1* using an antisense probe. Sections detected using a sense probe of *SlPIN1* (**m**–**o**) were used as controls. The insert in d shows a magnified image of the region shown in the white box. Tissues are indicated by white letters. C, cortex, V, vascular bundle, P, pith, AL, abscission layers. Scale bar = 100 μm.

Because the sensitivity to the abscission signal was altered in the AZ 4 h later after the flower removal^11^, *SlPIN1* expression in different tissues of the pedicel 0–4 h after flower removal was analysed. Longitudinal sections of pedicels were analysed using a *SlPIN1* mRNA specific probe by performing *in situ* hybridization. The hybridization signal in the proximal part was lower than that in the distal part and the AZ at 0 h, particularly in the pith (Fig. 2a–c). At 1 h after the flower removal, the signal prominently decreased in all three parts (Fig. 2d–f). In particular, some regions showed lower signals than the adjacent tissues, such as the cortex in the distal part and AZ (Fig. 2d, box). At 2 h, the *SlPIN1* expression further decreased in all three parts, while the cortex in the distal part recovered its expression (Fig. 2g–i). However, the cells in the abscission layer of the AZ showed a relatively higher expression at 1–2 h (Fig. 2e,g, AZ). At 4 h after the flower removal, a low hybridization signal was visible only in the AZ.

### Changes in the SlPIN1 protein level and protein phosphorylation in the AZ during abscission

**Figure 3:**
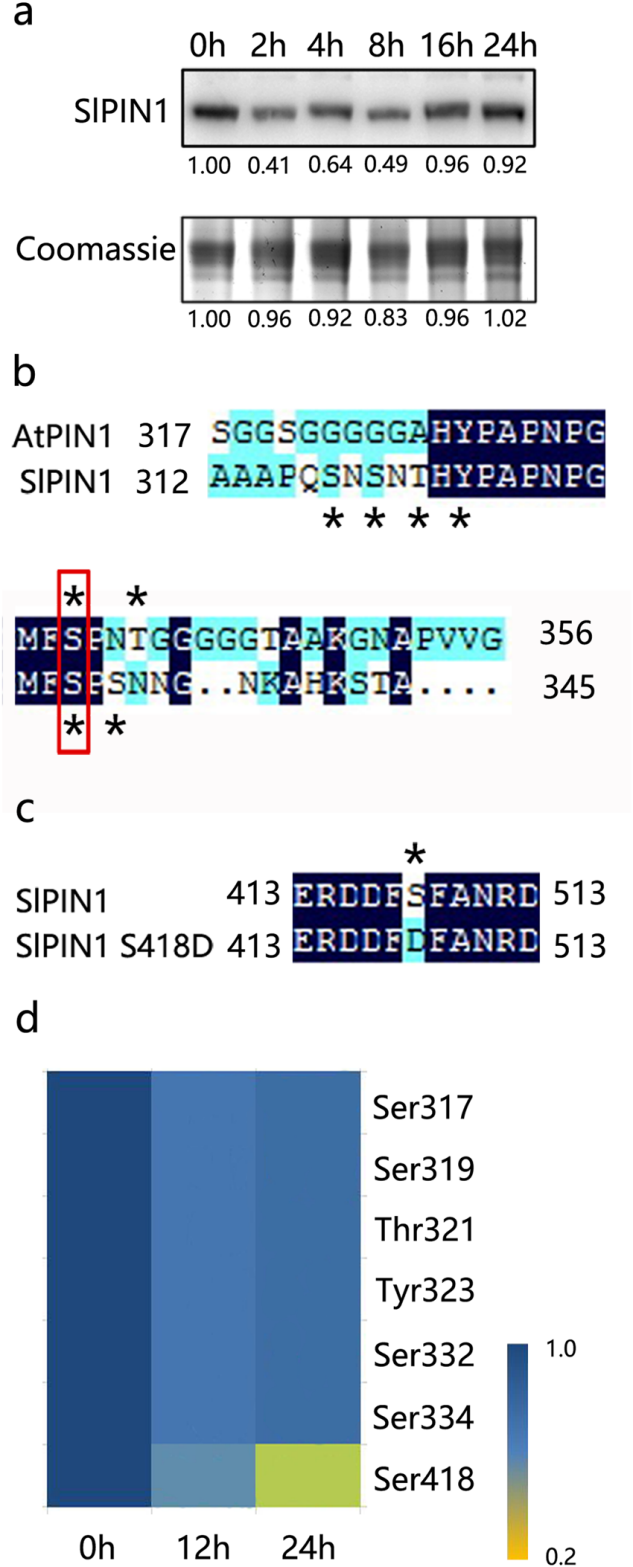
Changes in the SlPIN1 phosphorylation levels and protein accumulation in the AZ during abscission. (**a**) SlPIN1 protein levels were detected by western blotting in the AZ at 0 h, 2 h, 4 h, 8 h, 16 h and 24 h after flower removal. Coomassie-staining Rubisco protein is shown for total loading control (lower panel, full image is shown in Supplementary Figure S2). (**b**) Amino acid sequence alignment of AtPIN1 (317–356) and SlPIN1 (312–345) in the region containing the phosphorylation sites (asterisk). The framed Ser are conserved in the SlPIN1 and AtPIN1. (**c**) The phosphorylation site Ser418 (asterisk) in the amino acid sequence of SlPIN1 (413–513) was mutagenized to Asp. (**d**) SlPIN1 phosphorylation site status in the AZ 0 h, 12 h and 24 h after flower removal following ethylene treatment.

To further elucidate the possible mechanisms of SlPIN1 in abscission, the SlPIN1 protein level in the AZ was also analysed. A Western blot analysis was performed to determine the SlPIN1 protein abundance in the AZ using specific antibodies targeting SlPIN1. The SlPIN1 protein level in the AZ sharply decreased within 8 h, but returned to normal levels (compared with 0 h) 16–24 h after flower removal (Fig. 3a). In addition to the protein abundance, the phosphorylation of the PIN protein also plays an important role in PIN auxin transport^39,40,45^. Previously, we examined the changes in the phosphoproteomics of the AZ during abscission using the isobaric tag for relative and absolute quantification (iTRAQ) method, and seven phosphorylation sites of SlPIN1 (i.e., Ser317, Ser319, Thr321, Tyr323, Ser332, Ser334 and Ser418), corresponding to two phosphopeptides, were found^49^ (Fig. 3b–c, asterisks, the details are shown in the article by Zhang *et al*.). A conserved phosphorylation site, i.e., Ser337, which has been reported in AtPIN1 and other PM-localized AtPINs in *Arabidopsis* ^50^, was also found in SlPIN1 (Fig. 3b, red frame). When the pRS Score was ≥ 50 and Mascot Score was ≥ 20, a 1.5-fold abundance change was regarded as a significant change of phosphopeptides during abscission^49,51^. Among the seven sites, the phosphorylation levels at Ser418 were significantly decreased during abscission (Fig. 3d; Supplementary Table S2).

**Figure 4:**
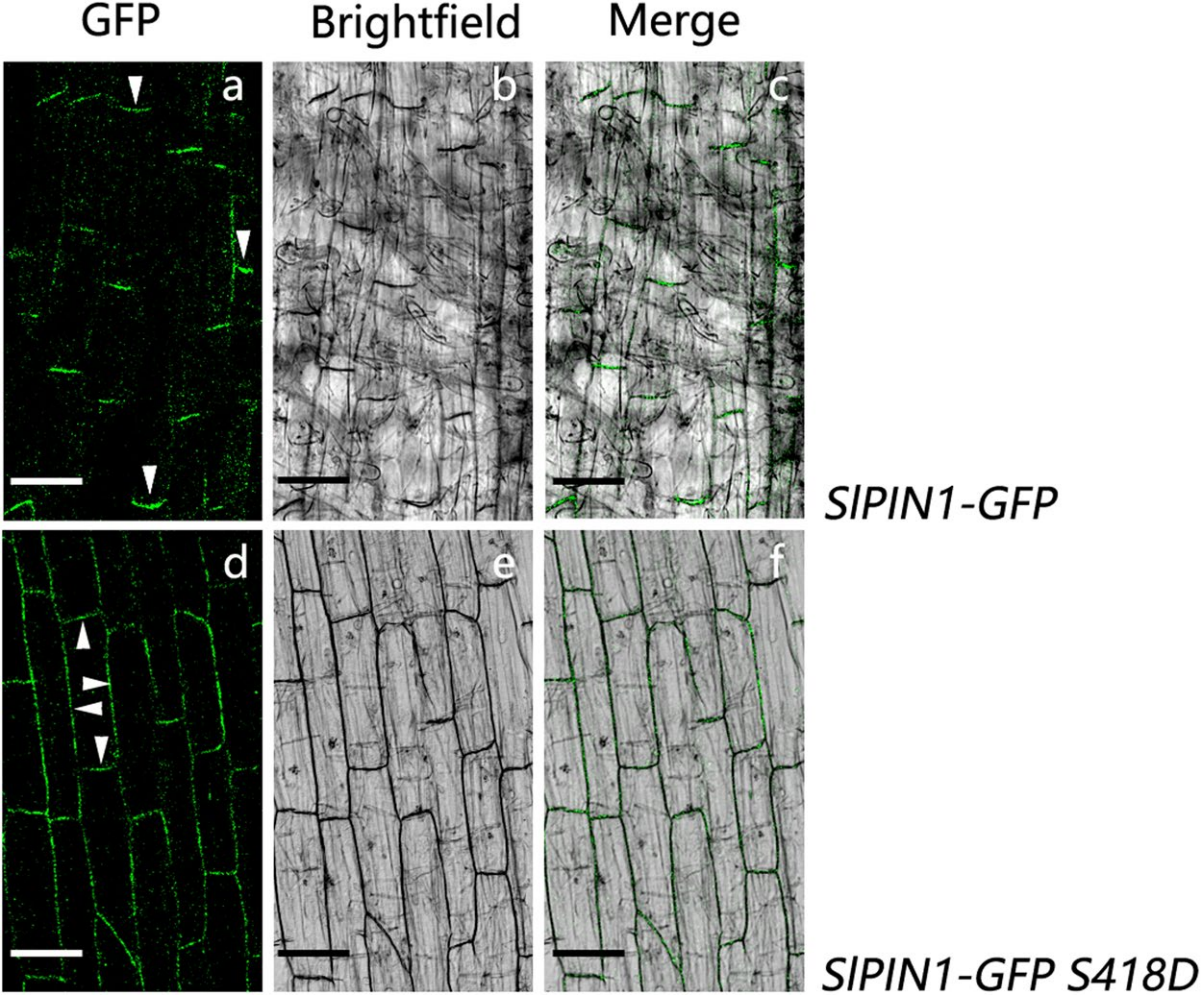
Subcellular localization of the SlPIN1-GFP protein and the phosphomimic SlPIN1-GFP S418D protein. GFP (**a**,**d**), bright-field (**b**,**e**) and merged (**c**,**f**) images were detected using a confocal laser scanning microscopy. (**a**–**c**) The basal localization of SlPIN1-GFP on the PM in tomato root cells. (**d**–**f**) Nonpolar localization of SlPIN1-GFP S418D on the PM in tomato root cells. The polarity is indicated by the white arrowheads. Scale bar = 50 μm.

To further understand the effect of phosphorylation at Ser418 on SlPIN1 polar localization, the construct *35 S::SlPIN1-GFP S418D* was generated from *35 S::SlPIN1-GFP* by replacing the Ser residue at site 418 (S418) with Asp (D) to mimic the phosphorylation status. The construct *35 S::SlPIN1-GFP* containing normal SlPIN1 protein was used as a control. A normally basal localization of SlPIN1-GFP was observed on the PM in the root cells (Fig. 4a–c). However, SlPIN1-GFP S418D was localized on the PM in a nonpolar manner in the same region of the root (Fig. 4d-f). Therefore, phosphomimicking at Ser418 induces the nonpolar localization of SlPIN1. Moreover, the decline of Ser418 phosphorylation levels during abscission may result in more basally localized SlPIN1 in the AZ.

### Analysis of the SIPIN1 interaction proteins in the AZ

**Figure 5:**
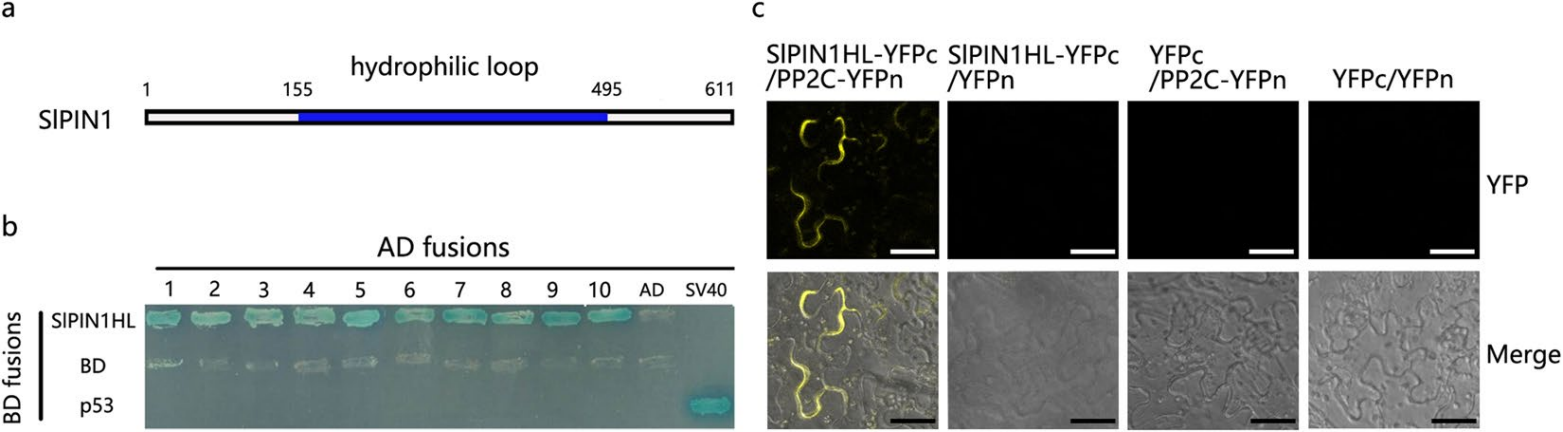
Interaction proteins of SlPIN1HL. (**a**) Structure of SlPIN1. SlPIN1HL was used as Y2H bait. (**b**) Interactions between SlPIN1HL and ten positive proteins were confirmed using Y2H assay. The ten interaction proteins are indicated by numbers 1 to 10. 1, PREDICTED: *Solanum lycopersicum* mitochondrial outer membrane protein porin of 34 kDa. 2, Glutamine synthetase. 3, PREDICTED: expansin-like B1. 4, proteasome-like protein alpha subunit-like. 5, PREDICTED: Kunitz-type protease inhibitor B (E3 ubiquitin-protein ligase). 6, PREDICTED: LRR receptor-like serine/threonine-protein kinase FEI 2 isoform X 1. 7, PREDICTED: LysM domain receptor-like kinase 4. 8, protein phosphatase 2 C. 9, ribonuclease T2 precursor. 10, pathogenesis-related protein STH-2. SV40 and P53 were used as positive controls. The AD and BD vectors were used as negative controls. The blue strain indicates the interaction between two proteins. (**c**) BiFC confirmation of the interaction between SlPIN1HL and PP2C. Fusion proteins of SlPIN1HL-YFPc, PP2C-YFPn, YFPn and YFPc were pairwise-expressed in tobacco leaves. YFPn and YFPc were used as negative controls. Scale bars = 50 μm.

To identify proteins that likely interact with SlPIN1, we used SlPIN1 with a hydrophilic loop (SlPIN1HL) as bait to screen a tomato pedicel abscission yeast cDNA library three times. The hydrophilic loop of SlPIN1 ranges from 155 aa to 495 aa as predicted by TMHMM Server v. 2.0. Suitable proteins were screened using the following two criteria: (1) a strong interaction with SlPIN1HL as assessed by a yeast two-hybrid (Y2H) assay and (2) a high frequency of possible proteins that could interact with SlPIN1HL as indicated by the Y2H screening. Ten proteins were identified to interact with SlPIN1HL (Table 1 and Fig. 5a,b). Among these proteins, proteasome-like protein alpha subunit-like and Kunitz-type protease inhibitor B participated in the ubiquitin-dependent degradation process, and the LRR receptor-like serine/threonine-protein kinase FEI 2 isoform × 1, the LysM domain receptor-like kinase 4 and protein phosphatase 2 C were involved in protein phosphorylation. Bimolecular fluorescence complementation (BiFC) analysis further confirmed the interaction between SlPIN1HL and PP2C (Fig. 5c), of which gene expression was reported to be abscission-specifically activated during pedicel abscission^11^.

**Table 1:** List of ten interactive proteins with SIPIN1HL by Y2H screening during abscission.

Number	Name	Number of amino acids	Frequency^a^	Accession number
1	PREDICTED: *Solanum lycopersicum* mitochondrial outer membrane protein porin of 34 kDa	276	2	SGN-U570210
2	Glutamine synthetase	432	4	SGN-U578728
3	PREDICTED: expansin-like B1	253	3	SGN-U582695
4	proteasome-like protein alpha subunit-like	249	2	SGN-U577617
5	PREDICTED: Kunitz-type protease inhibitor B (E3 ubiquitin-protein ligase)	334	1	SGN-U567148
6	PREDICTED: LRR receptor-like serine/threonine-protein kinase FEI 2 isoform X 1	589	3	SGN-U564473
7	PREDICTED: LysM domain receptor-like kinase 4	654	2	SGN-U564297
8	protein phosphatase 2 C	282	4	SGN-U581169
9	ribonuclease T2 precursor	230	1	SGN-U580032
10	pathogenesis-related protein STH-2	160	1	SGN-U578441

^a^The number of times the protein interacted with SlPIN1 during the Y2H screening process.

### Suppressed expression of *SlPIN1* accelerates tomato pedicel abscission

**Figure 6:**
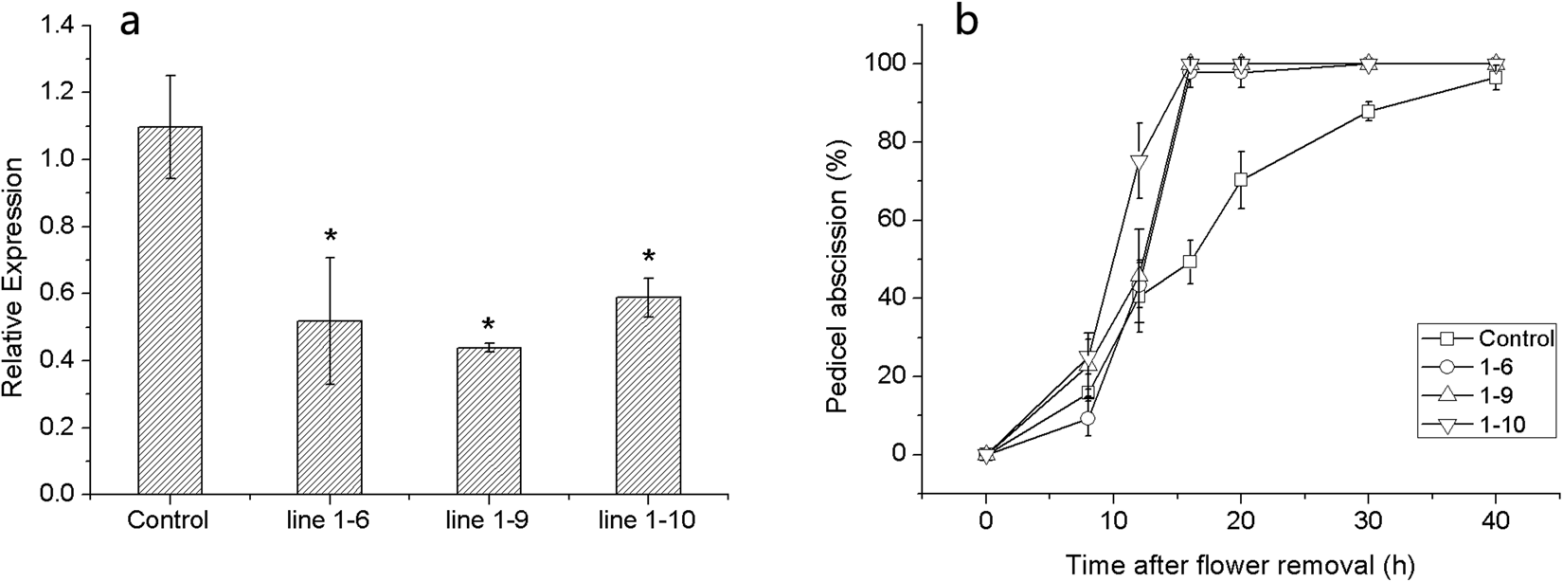
VIGS decreases *SlPIN1* expression and accelerates pedicel abscission. (**a**) *SlPIN1* expression in the flowers during the anthesis of the *SlPIN1*-VIGS lines (1–6, 1–9 and 1–10) and *DR5*::*GUS* (Control). *SlPIN1* expression decreased by approximately 50% in the *SlPIN1*-VIGS lines. The data are presented as the mean (±SD) corresponding to three independent tests. The asterisk indicates significant differences (*P* < 0.05) according to Duncan’s multiple range test. (**b**) Pedicels in the *SlPIN1*-VIGS lines (1–6, 1–9 and 1–10) abscised earlier than those in *DR5*::*GUS* (Control) after flower removal. The data are presented as the mean (±SD) corresponding to three independent tests, and 20 to 30 pedicels were investigated in each test.

**Figure 7:**
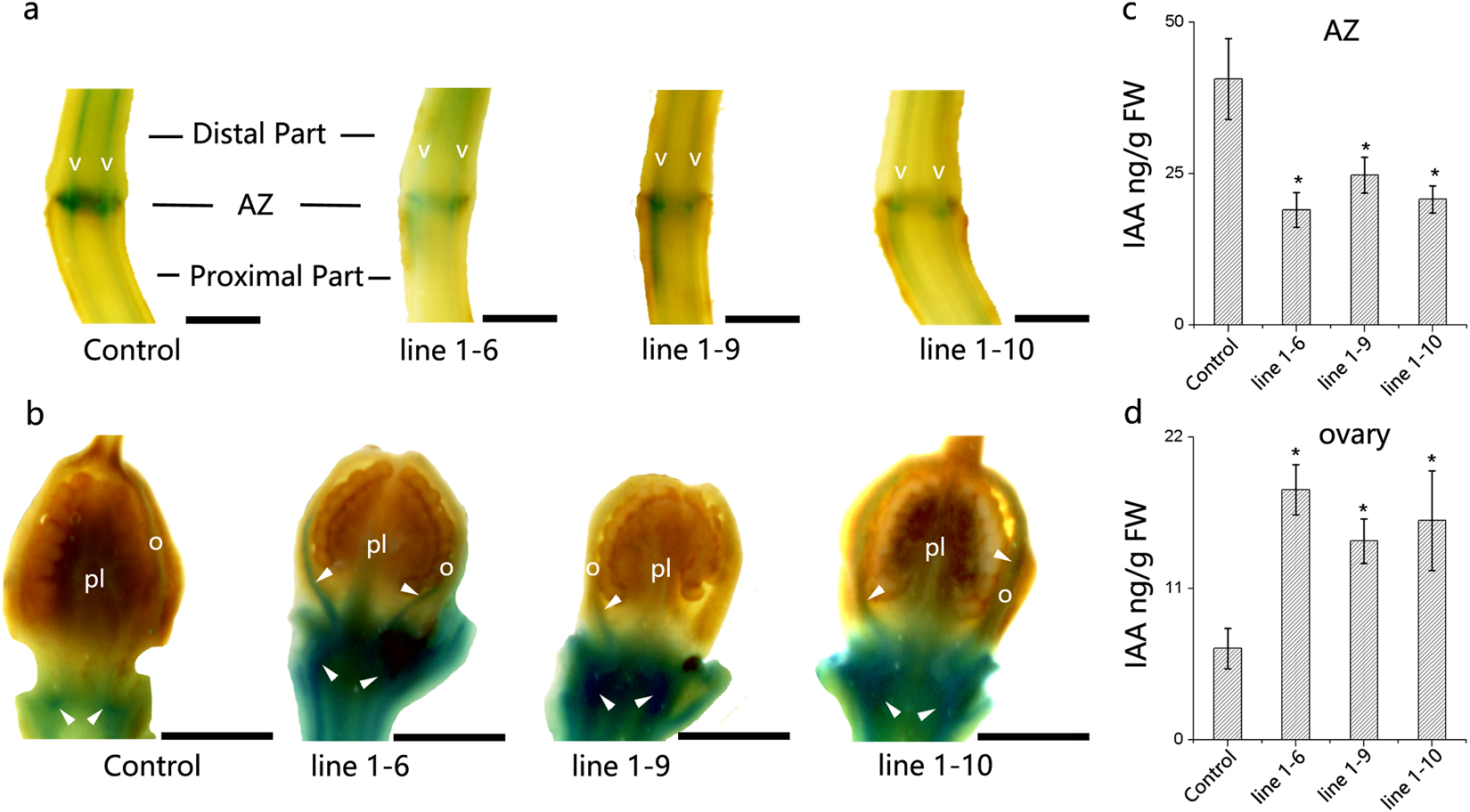
GUS expression in the *SlPIN1*-VIGS line. (**a**) GUS staining in the pedicel. GUS staining is observed in the vascular strands (v) and the AZ of the pedicel, and the GUS staining in the Control is much deeper than that in line 1–6, 1–9 and 1–10. (**b**) GUS staining in the ovary. Strong GUS staining is observed in the ovary wall (o) and the basal tissue of the ovary in line 1–6, 1–9 and 1–10 (arrowheads). Only slight staining is visible in the basal tissue connecting the pedicel in the Control (arrowheads). The IAA levels in the AZ (**c**) and ovary (**d**) are shown in histograms. The asterisk indicates significant differences (*P* < 0.05) according to Duncan’s multiple range test. Flowers were sampled at anthesis. pl, placenta, o, ovary wall. Scale bars = 1 mm.

To investigate the function of *SlPIN1* in the pedicel abscission process, silencing of a transgenic line of *SlPIN1* was performed by VIGS in the *DR5::GUS* auxin reporter line. A *DR5::GUS* line transformed with an empty vector was used as the control. β-glucuronidase (GUS), which was promoted by the auxin responsive promoter, i.e., *DR5*, reflected the auxin distribution in the *DR5::GUS* auxin reporter line^52^. Ten independent transgenic plants were obtained, and three *SlPIN1*-VIGS lines (1–6, 1–9 and 1–10) showed significantly (*P* = 0.008, 0.002 and 0.005, respectively) suppressed *SlPIN1* expression in the flower, which was approximately 50% of the normal (Control) expression level (Fig. 6a). To investigate the involvement of *SlPIN1* in abscission, *SlPIN1-*VIGS lines 1–6, 1–9 and 1–10 were selected to test the abscission rate. All three *SlPIN1*-VIGS lines showed accelerated abscission processes, and almost all pedicels abscised at 16 h, while the abscission rate of *DR5::GUS* was 50% (Fig. 6b). To investigate the changes in auxin transport and distribution, GUS activity was detected. In the *DR5::GUS* pedicels, prominent GUS staining was observed in the AZ and vascular strands, and this staining was stronger in the distal part than in the proximal part (Fig. 7a, Control), which was consistent with previous auxin distribution observations^15^. However, GUS activity in the *SlPIN1*-VIGS lines was much weaker in the AZ and vascular strands than in *DR5::GUS* (Fig. 7a). The auxin accumulation in the ovary, which is the source of IAA^7,10^, was also analysed. In the *SlPIN1*-VIGS lines, strong GUS activity was detected in the basal tissue connecting the pedicel, and the ovary wall also showed intermittent GUS staining (Fig. 7b, line1–6, line1–9 and line 1–10, arrowheads). In the normal *DR5::GUS* plants, only slight GUS staining was visible in the basal tissue connecting the pedicel (Fig. 7b, Control, arrowheads), and no GUS activity was observed in the ovary.

## Discussion

The expression of *SlPIN1*, which is an auxin efflux facilitator, in the distal part and AZ was prominently higher than that in the proximal part (Fig. 1b–d, 0 h; Fig. 2a–c), which was consistent with the basipetal auxin flux in the tomato pedicel^12^. *PIN1* was previously reported to be regulated by auxin^26,28,29,53^, and certain *Aux/IAAs* and *ARFs* have been shown to regulate the expression of *PIN* genes^29,31^. We also detected certain putative AuxREs in the *SlPIN1* promoter sequences (TGTCTC, TGTCCC). After the IAA source was removed by cutting the flowers, the expression levels in all parts of the pedicel sharply decreased (Fig. 1 0–4h; Fig. 2), revealing the high sensitivity of *SlPIN1* to auxin depletion. This sensitivity was supported by the result of the IAA treatment, which dramatically increased the *SlPIN1* expression throughout the pedicel (Fig. 1c–d; Supplementary Fig. S1b). *SlPIN1* also showed a tissue-specific expression pattern at 0-4 h. The lower expression in the cortex of the distal part and the AZ indicates a higher sensitivity to auxin decline in this region, suggesting that the cortex may not be the main function region of *SlPIN1*. The AZ maintained a relatively higher level of *SlPIN1* expression, suggesting that high auxin transport activity occurs in this area.

*SlPIN1* expression in the AZ was slightly up-regulated after 4 h and reached approximately one-third of its normal levels approximately 24 h after the onset of abscission (Fig. 1). In contrast, the SlPIN1 protein levels in the AZ were considerably increased and reached their normal levels after 8 h (Fig. 3a). Although the increase in SlPIN1 was found at both the gene and protein levels during abscission, the changes in the two levels were not consistent with each other. This difference indicates that in addition to transcription and translation, the abundance of the SlPIN1 protein may be influenced by other levels of mediation. Ubiquitination and proteasome activity have been previously reported to be involved in AtPIN2 turnover and auxin redistribution in the gravitropic response process, and the proteasome inhibitor MG132 enhances the stabilization and abundance of AtPIN2^42,54^. In this study, SlPIN1 interacted with two proteins associated with ubiquitination and proteasome activity, suggesting that a ubiquitin-dependent pathway may be involved in SlPIN1 degradation. Therefore, the delayed degradation of SlPIN1 could increase the stabilization of the SlPIN1 protein in the AZ, which could enhance auxin transport in the AZ after auxin depletion.

PIN1 phosphorylation is involved in auxin-mediated plant growth and development^39,40^. The serine residues in the TPRXS motif and the Ser337/Thr340 residues in AtPIN1 are conserved phosphorylation sites found in *Arabidopsis* ^38,45,50^. The TPRXS motif, but not Ser337/Thr340, is recognized and phosphorylated by PID, WAG1 and WAG2^38,45^. The same conserved site was also detected in SlPIN1 (Ser332, Fig. 3b), while other sites of SlPIN1 were found uniquely. In addition, the interaction proteins of SlPIN1 found during abscission (i.e., LRR receptor-like serine/threonine-protein kinase FEI2 isoform X 1 (FEI2), LysM domain receptor-like kinase and protein phosphatase 2 C (PP2C)) might be the potential kinases and phosphatase of SlPIN1; however, further experiments are needed to confirm this hypothesis. The phosphorylation sites, kinase and phosphatase of SlPIN1 newly discovered in our study reveal the complexity of PIN protein phosphorylation. *PP2C* (a potential phosphatase of SlPIN1) was activated during pedicel abscission, and IAA suppressed this activation, whereas ethylene promoted it, which is assumed as a abscission related gene^11^. We also found a down-regulated expression pattern of *FEI2* (a potential kinase of SlPIN1) during abscission in the AZ, whereas this down-regulation was inhibited by 1-MCP treatment (Supplementary Figure S3). The decrease of a kinase and the increase of a phosphatase during abscission may induce SlPIN1 to be dephosphorylated. In fact, phosphorylation of SlPIN1 at Ser418 decreased during abscission (Fig. 3d; Supplementary Table S2). Phosphorylation influences the subcellular localization of PIN and the alteration in auxin transport and distribution. Our results showed that phosphorylation at Ser418 could reduce the basal localization of SlPIN1 (Fig. 4), which is consistent with the observations mimicking phosphorylation at AtPIN1 Ser337/Thr340^50^. The basal localization and roles of the basipetal auxin transport of PIN1 have been reported in *Arabidopsis* ^4^ and tomato^1^. SlPIN1 phosphorylation at Ser418 was significantly decreased during abscission, indicating that more SlPIN1 proteins localize basally on the PM in the AZ. Altogether, these results indicate that during the later stages of abscission, SlPIN1 may enhance the basipetal auxin transport and accelerate the auxin decrease in the AZ.

After the flower removal, the increase in SlPIN1 and its basal localization in the AZ suggests that SlPIN1 may promote the basipetal auxin flux and auxin decrease in the AZ, which contributes to pedicel abscission. In addition, the reduced expression of *SlPIN1* in the VIGS lines accelerated abscission. Premature abscission was observed in transgenic lines with low IAA levels in the AZ of *Arabidopsis*, suggesting that the auxin levels play a key role in the regulation of organ abscission^14^. With the constant basipetal auxin flux through the AZ, the relatively high level of auxin prevents the AZ from becoming sensitized to ethylene and abscising^55–58^. The *SlPIN1* VIGS lines showed reduced IAA levels in the AZ in the tomato pedicel, which may be the reason of the accelerated abscission. In addition, the suppression of *SlPIN1* induced an abnormal auxin accumulation in the ovary and the basal tissues that connect the ovary and the pedicel, but reduced auxin levels were observed in the vascular strands and the AZ in the pedicel, indicating that silencing *SlPIN1* causes basipetal auxin transport defects in the flower and pedicel. *SlPIN1* have been reported to be expressed preferentially in the placenta during fruit development, indicating that SlPIN1 plays a role in regulating the basipetal auxin flux from the seed to the plant^7^. Our results reveal that SlPIN1 plays a major role in the source-sink transport of auxin from the fruit to the basal organ, and silencing its expression could decrease the AZ auxin content, which is essential for the prevention of abscission.

The effect of auxin activation on the SlPIN1 gene and the major role of SlPIN1 on basipetal auxin transport during pedicel abscission were described above. Ethylene is one of the two most important hormones in the regulation of abscission and often acts directly during the late stage of abscission execution and defence layer formation^11^. As a result of crosstalk between ethylene and auxin, ethylene inhibits root elongation and lateral root initiation by enhancing IAA transport and accumulation in the root, which is achieved by increasing the expression of the *PIN* genes in the root^34,35^. We also found ethylene response factor (ERF) binding sites in the *SlPIN1* promoter (GCCGCC), indicating that *SlPIN1* may also be regulated by ethylene. Interestingly, the up-regulation of *SlPIN1* in the AZ and proximal part occurred during the late phase of tomato flower abscission, during which the AZ is sensitive to ethylene^11^. The ethylene inhibitor 1-MCP delayed the up-regulation of *SlPIN1* expression (Fig. 1, 1-MCP), suggesting that ethylene may up-regulate *SlPIN1* to accelerate auxin transport in order to accelerate auxin decrease in the AZ and promote the abscission process. In addition, the up-regulation of *PP2C* ^11^ and the down-regulation of *FEI2* (Supplementary Figure S3) at the late stage of abscission were inhibited by 1-MCP treatment, implying that ethylene could enhance SlPIN1 dephosphorylation by promoting *PP2C* expression and suppressing *FEI2* transcription. Hence, ethylene could promote the basipetal auxin transport of SlPIN1 by influencing its phosphorylation status. Our results indicate that ethylene might affect SlPIN1 at both the transcriptional and posttranslational levels during abscission.

**Figure 8:**
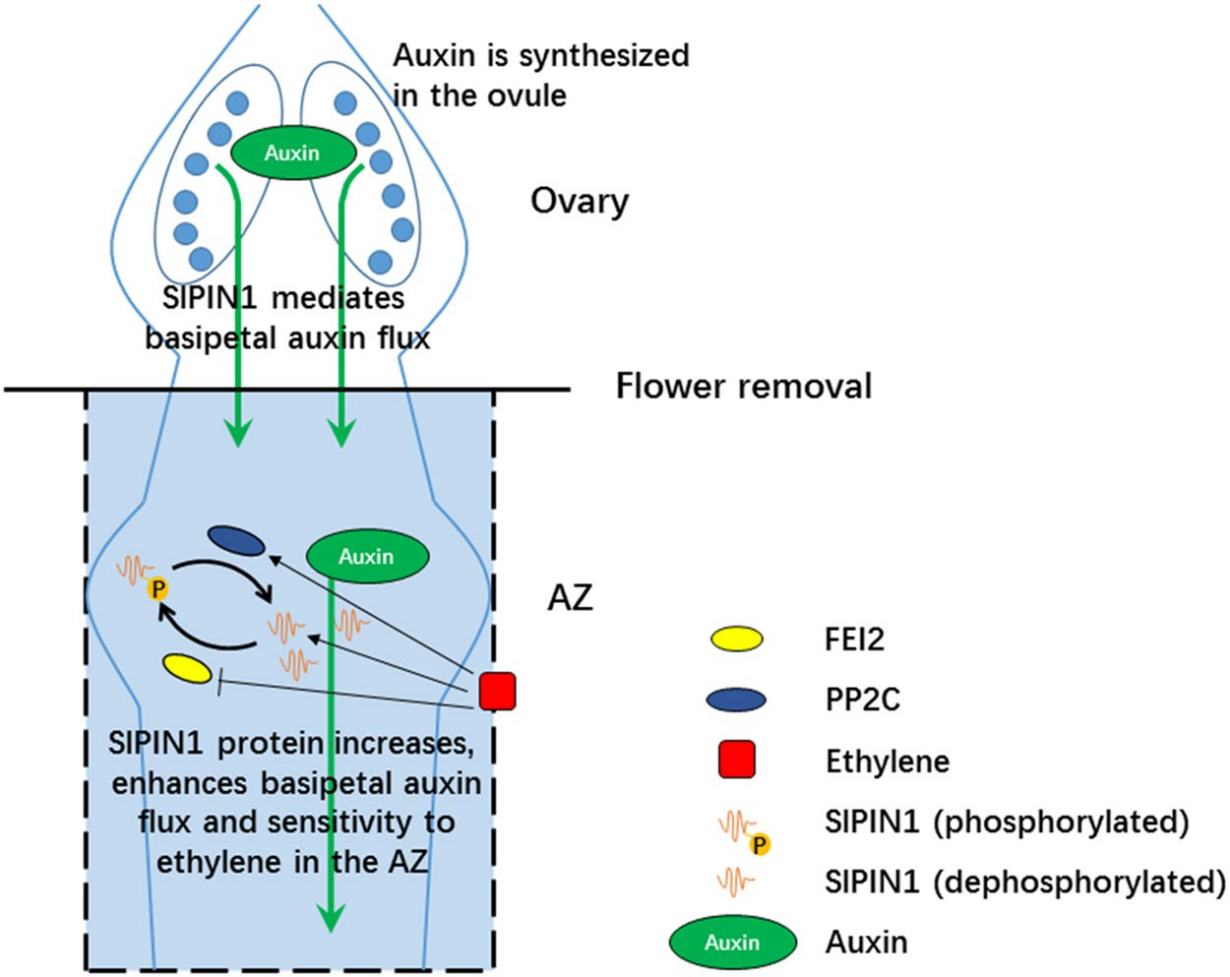
A schematic depiction illustrating a major role of SlPIN1 of basipetal auxin transport in flower organ and a postulated relationship among SlPIN1, auxin and ethylene in the regulation of pedicel abscission. SlPIN1 plays a major role in mediating auxin source-sink transport from ovary to basal organ. Auxin depletion induces SlPIN1 decrease after flower removal, but SlPIN1 protein levels in the AZ increase from 8 h after flower removal. In addition, the decreased phosphorylation levels during abscission may result in basal PM-localization of SlPIN1 protein in the AZ, which may result in improved basipetal auxin flux and sensitivity to ethylene in the AZ. Ethylene may regulate SlPIN1 auxin transport capability by improving *SlPIN1* expression during abscission. In addition, ethylene influences *FEI2* and *PP2C* expression during abscission, which are the potential kinase and phosphatase of SlPIN1, implying ethylene may also affect SlPIN1 phosphorylation status.

Taken together, our results in this work support a model (Fig. 8) that SlPIN1 plays a major role in regulating basipetal auxin transport from ovary to basal tissues. And the improvement of SlPIN1 protein levels and protein dephosphorylation levels in the AZ during abscission indicates that SlPIN1 may also play a role in abscission process by enhancing basipetal auxin flux. In addition, ethylene affects the expression of *SlPIN1* and two interaction proteins of SlPIN1, implying an involvement of ethylene in the regulation of auxin transport during abscission.

## Methods

### Plant materials and treatments

Tomato cultivars (*Solanum lycopersicum* L. cv Zhongshu 6) were grown in soil for 7 weeks in a greenhouse (25 ± 3 °C during the day and 15 ± 3 °C during the night) with natural light. The *DR5::GUS* and *SlPIN1* VIGS transgenic lines were grown in soil in an artificial climate chamber (25 °C during the day and 15 °C during the night) with natural light. The pedicel explants were obtained from the tomato flowers during anthesis by removing the petals and base. After the proximal end was inserted into 1% w/v agar medium, the pedicels were incubated in a glass container (20 L) at 25 °C. For the IAA treatment, the pedicels were inserted into agar medium containing 50 μg/g IAA. For the 1-MCP treatment, the pedicel explants were exposed to 1-MCP (20 μL/L) in a container. The pedicel explants were sampled at different times after the flower removal according to the different experiments (0 h, 2 h, 4 h, 8 h, 16 h and 24 h for the RT-PCR experiment; 0 h, 1 h, 2 h and 4 h for the *in situ* hybridization experiment; 0 h, 8 h, 16 h and 24 h for the Western blot analysis; and 0 h, 4 h, 8 h, 16 h and 24 h for the construction of the cDNA library). For tissue sampling, the pedicels were cut into less than 1 mm sections at the joint position in the AZ tissue or 3 mm at the distal and proximal side adjacent AZ. The tissue samples were placed in liquid nitrogen immediately and frozen at −80 °C until the RNA and protein extraction. The abscission rates were recorded as described by Wang *et al*.^59^.

### RNA extraction and RT-PCR

RNA was extracted using an RNAprep pure plant total RNA extraction kit (Qiagen, Germany), and contaminating genomic DNA was removed by DNase I. RT-PCR was performed according to Jain *et al*.^60^. In brief, cDNA was used as a template and mixed with *SlPIN1* primers (Supplementary Table S1) and the SYBR Premix Ex Taq (Takara, Japan) for qRT-PCR analysis using the ABI 7500 Real Time PCR system (Applied Biosystems, USA). The reaction conditions were as follows: 95 °C for 30 s, 40 cycles of 95 °C for 5 s and 60 °C for 34 s, 1 cycle of 95 °C for 15 s, 60 °C for 1 min and 95 °C for 15 s. *26 S* ribosomal RNA was used as an internal reference^61^. The Ct values of the target genes were normalized to the Ct value of *26 S* ribosomal RNA, and the relative expression levels were calculated using the 2^−ΔΔCt^ method^62^. At least three biological replicates of independent RNA isolations were subjected to RT-PCR and all results were presented as the means ± standard deviation (SD).

### Statistical analysis

All results were presented as the means ± standard deviation (SD). The statistical analyses of the results were performed using SPSS 17.0 (SPSS Inc., USA). Significant differences were determined using Duncan’s multiple range test at a level of *P* < 0.05.

### Probe synthesis and *in situ* hybridization

For the DIG-labelled probe synthesis, the *in vitro* transcription vector pSPT19 was constructed using a *SlPIN1-*specific fragment amplified using the primers described in Supplementary Table S1. The sense and antisense probes were synthesized through *in vitro* transcription using SP6 and T7 RNA polymerase according to the manufacturer’s protocol (DIG RNA Labeling Kit (SP6/T7), Roche, Germany). At 0, 1, 2 and 4 h after the flower removal, the pedicel explants were collected, fixed in a 4% paraformaldehyde solution (Phygene, China) at 4 °C for at least 16 h, dehydrated and embedded in paraffin according to Pirttilä^63^. The protocol for the *In situ* hybridization was modified according to Abcam (http://www.abcam.com/protocols/ish-*in-situ*-hybridization-protocol). Longitudinal 8 μm slices were cut using a Microtome Leica RM2235 (Leica, Germany), dewaxed with xylene and rehydrated in ethanol gradient solutions. After the treatments with proteinase K and acetic acid, the slices were washed with saline sodium phosphate EDTA (SSPE) and dehydrated in ethanol gradient solutions. Then, the slices were incubated in a hybridization solution containing a *SlPIN1* probe at 55 °C overnight. After washing with saline-sodium citrate (SSC) gradient solutions, the slices were blocked with a blocking reagent (Roche, Germany) and incubated with anti-digoxigenin-AP (Roche, Germany) for 1 h at room temperature. Finally, the slices were stained with NBT/BCIP (Roche, Germany) at 37 °C overnight. The samples were then examined under a Nikon Eclipse 80i light microscope (Nikon Japan).

### Antibodies and Western blot analysis

A primary antibody targeting SlPIN1 was generated by the Abmart Company using a synthetic peptide (NFGANDVYGMSNNS) derived from SlPIN1. The anti-β-actin and secondary antibodies were obtained from the Kangwei Company. The total protein in the AZ at 0, 8, 16 and 24 h was extracted using the Minute™ total protein extraction kit (Invent Biotechnologies, USA). After boiling in a water bath for 5 min and separating via 10% SDS-PAGE, the protein samples were transferred onto polyvinylidene difluoride (PVDF) membranes using the Trans-BlotTurbo™ (Bio-Rad, USA) transfer system. Then, the PVDF membranes were incubated in 5% BSA at room temperature for 2 h with a primary antibody at 4 °C overnight and a second antibody at room temperature for 1 h. Finally, the DAB Horseradish Peroxidase Color Development Kit was used for chemical coloration on the Azure c600 Western blot imaging system (Azure, USA).

### Phosphorylation site assay

Pedicels were exposed to ethylene (20 μL/L) and sampled at time points of 0 h, 12 h and 24 h. The total protein was extracted and digested, and then labelled using the 4-plex iTRAQ reagent (Applied Biosystems, USA). To enrich the phosphorylated peptides, the samples were mixed with TiO_2_ beads. All the iTRAQ-labelled peptides were analysed using a Q Exactive mass spectrometer that was coupled to Easy nLC (Thermo Fisher Scientific, USA). Phosphorylated peptides were analysed using Proteome Discoverer 1.4 (Thermo Electron, USA). The method has previously been described in detail^49^.

### Construction of vectors and plant transformation

The full-length cDNA of *SlPIN1* was synthesized and cloned into the vector pCAMBIA1300-35S-GFP (p35S::SlPIN1-GFP). The construct p35S::SlPIN1-GFP S418D was generated from p35S::SlPIN1-GFP by converting Ser418 (AGC) of PIN1 to Asp (GAC) by site-directed mutagenesis. Both constructs were generated by the Transduction Bio Company. The sequence verified constructs were introduced into *Agrobacterium tumefaciens* LBA4404 by electroporation. Seeds of Zhongshu 6 were germinated in the dark at 28 °C for 4 days. The roots were then permeated with a suspension liquid of *A*. *tumefaciens* LBA4404 using a vacuum pump for 15 min and cultured for 2 days in the dark at 25 °C before subcellular analysis.

VIGS of *SlPIN1* was performed using *Tobacco rattle virus* (TRV)-based vectors (pTRV1/2)^64^. The specific fragment of *SlPIN1* was PCR amplified using primers described in Supplementary Table S1, digested with EcoRI and BamHI and cloned into the vector pTRV2. The pTRV1 and pTRV2 vectors were transformed into *Agrobacterium tumefaciens* GV3101. The suspension liquid of GV3101 containing pTRV1 and pTRV2 was mixed at a ratio of 1: 1, and the mixture was infiltrated into the *DR5::GUS* seedlings (3-leaves stage, 40 days old). A mixture of pTRV1 and an empty pTRV2 was used as a control. The plants were subsequently grown in an artificial climate chamber under previously described conditions until the anthesis stage (approximately 42 days). For the silencing efficiency analysis, the whole flowers at anthesis were sampled and detected by RT-PCR.

### Subcellular localization analysis

Subcellular localization analysis was performed according to Sakamoto & Briggs^65^. In brief, after culturing for 2 days in the dark, roots from transformed seedlings were placed on cover slips, and GFP fluorescence was detected using a Leica SP8 confocal microscope (Leica, Germany).

### GUS analysis

The flower samples were incubated in GUS substrate solution containing 50 mM sodium phosphate, pH 7, 1 mM K_3_/K_4_ FeCN, 0.1% (w/v) Triton X-100, and 2 mM 5-bromo-4-chloro-3-indolyl-b-GlcUA (Duchefa Biochemie, The Netherlands) for 8 h (pedicel) or 48 h (ovary). Then, the samples were washed once with water and incubated in an ethanol/ acetic acid (1: 3) solution at 55 °C for 20 min. The samples were imaged using Epson lmageScanner III (Epson, Japan).

### IAA quantification by ELISA

The extraction and quantification of IAA from the ovary and AZ samples were performed according to Yang *et al*. and He^66,67^. The IAA standards, antigens and antibodies against IAA were obtained from Phytohormones Research Institute (China Agricultural University). The samples were homogenised and extracted in cold 80% methanol with 1 mmol/L butylated hydroxytoluene at 4 °C for 4 h. After centrifugation at 4000 rpm for 15 min (4 °C), the extracts were passed through a C18 Sep-Pak cartridge (Waters, USA), dried in N_2_, and dissolved in 2 mL of PBS (pH 7.5) with 0.1% (v/v) Tween 20 and 0.1% (w/v) gelatine. The 96-well microfiltration plates were coated with antigen against IAA in NaHCO_3_ buffer (50 mmol/L, pH 9.6) and incubated overnight at 4 °C. The microfiltration plates were washed four times with PBS (pH 7.5) with 0.1% (v/v) Tween 20, and each well was filled with 50 μL extracts or IAA standards, and 50 μL antibodies against IAA. The plates were incubated at 37 °C for 30 min and washed as above. Then, IgG horseradish peroxidase was added to each well, incubated for 30 min at 37 °C and washed. Finally, the substrate (ortho-phenylenediamine) was added and incubated for 30 min at 37 °C. The absorbance at 490 nm was detected using Infinite M200pro (Tecan, Switzerland).

### Y2H assays

The construction of the tomato flower pedicel abscission cDNA library was accomplished according to the Make Your Own “Mate & Plate™” Library (Clontech). Total RNAs were extracted from the tomato pedicel AZ *in vitro* using the TaKaRa MiniBEST Universal RNA Extraction Kit (TaKaRa) and used for the cDNA library construction. The hydrophilic loop of SlPIN1 was predicted by TMHMM Server v. 2.0 (http://www.cbs.dtu.dk/services/TMHMM/). The *SlPIN1* hydrophilic loop (*SlPIN1*HL) fragment was amplified using primers for the bait construction in Y2H based on the *SlPIN1* full fragment as the template, digested with EcoRI and BamHI and cloned into vector pGBKT7 as the bait construction. The screening of the tomato flower pedicel abscission cDNA library and the confirmation of positive interactions were performed according to the Matchmaker™ Gold Yeast Two-Hybrid System User Manual (Clontech). Yeast plasmids that interacted with SlPIN1HL-pGBKT7 were extracted using Zymoprep І, Yeast Plasmid Miniprep (ZYMO RESEARCH). The extracted yeast plasmids were then sequenced and searched for in the National Center for Biotechnology Information (https://www.ncbi.nlm.nih.gov/) and Sol Genomics Network (https://solgenomics.net/). Different clones for each combination were co-transformed into the Y2HGold yeast strain and grown on SD/–Leu/–Trp medium and SD/–Ade/–His/–Leu/–Trp/X-a-Gal/AbA medium at 30 °C for 72 h for the interaction detection.

### BiFC assay

BiFC assay analysis was performed as previously described^68^. In brief, full-length coding sequences of SlPIN1HL and PP2C without their stop codons were subcloned into pCambia1300-35S-CYFPc (YFPc) and pCambia1300-35S-CYFPn (YFPn) vectors. These constructs were transformed into *A*. *tumefaciens* EHA105 and then co-expressed into tobacco leaves. Transfected tobacco leaf epidermal cells were imaged for YFP fluorescence using a Leica SP8 confocal microscope (Leica, Germany).

### Data availability

All data generated or analysed during this study are included in this published article (and its Supplementary Information files).

## Electronic supplementary material

Supplementary Information

## References

[1] Ivanchenko MG (2015). The cyclophilin A DIAGEOTROPICA gene affects auxin transport in both root and shoot to control lateral root formation. Development.

[2] Mignolli F, Mariotti L, Picciarelli P, Vidoz M (2017). Differential auxin transport and accumulation in the stem base lead to profuse adventitious root primordia formation in the aerial roots (aer) mutant of tomato (Solanum lycopersicum L.). Journal of Plant Physiology.

[3] Reinhardt D, Pesce E-R, Stieger P, Mandel T (2003). Regulation of phyllotaxis by polar auxin transport. Nature.

[4] Gälweiler L (1998). Regulation of polar auxin transport by AtPIN1 in Arabidopsis vascular tissue. Science.

[5] Scarpella E, Marcos D, Friml J, Berleth T (2006). Control of leaf vascular patterning by polar auxin transport. Genes & development.

[6] Mattsson J, Ckurshumova W, Berleth T (2003). Auxin signaling in Arabidopsis leaf vascular development. Plant Physiology.

[7] Pattison RJ, Catalá C (2012). Evaluating auxin distribution in tomato (Solanum lycopersicum) through an analysis of the PIN and AUX/LAX gene families. The Plant Journal.

[8] Srivastava A, Handa AK (2005). Hormonal regulation of tomato fruit development: a molecular perspective. Journal of plant growth regulation.

[9] Sorefan K (2009). A regulated auxin minimum is required for seed dispersal in Arabidopsis. Nature.

[10] Petrášek J, Friml J (2009). Auxin transport routes in plant development. Development.

[11] Meir S (2010). Microarray analysis of the abscission-related transcriptome in the tomato flower abscission zone in response to auxin depletion. Plant Physiology.

[12] Taylor JE, Whitelaw CA (2001). Signals in abscission. New Phytologist.

[13] Estornell LH, Agustí J, Merelo P, Talón M, Tadeo FR (2013). Elucidating mechanisms underlying organ abscission. Plant Science.

[14] Basu MM (2013). The manipulation of auxin in the abscission zone cells of Arabidopsis flowers reveals that indoleacetic acid signaling is a prerequisite for organ shedding. Plant Physiology.

[15] Guan X (2014). Temporal and spatial distribution of auxin response factor genes during tomato flower abscission. Journal of plant growth regulation.

[16] Meir S (2011). *Identification of defense-rel*ated genes newly-associated with tomato flower abscission. Plant signaling & behavior.

[17] Bar-Dror T (2011). Programmed cell death occurs asymmetrically during abscission in tomato. The Plant Cell.

[18] Friml J, Vieten A, Sauer M, Weijers D (2003). Efflux-dependent auxin gradients establish the apical-basal axis of Arabidopsis. Nature.

[19] Kaneda M (2011). ABC transporters coordinately expressed during lignification of Arabidopsis stems include a set of ABCBs associated with auxin transport. Journal of Experimental Botany.

[20] Friml J (2002). AtPIN4 mediates sink-driven auxin gradients and root patterning in Arabidopsis. Cell.

[21] Friml J, Wiśniewska J, Benková E, Mendgen K, Palme K (2002). Lateral relocation of auxin efflux regulator PIN3 mediates tropism in Arabidopsis. Nature.

[22] Wiśniewska J (2006). Polar PIN localization directs auxin flow in plants. Science.

[23] Bainbridge K (2008). *Auxin influx carriers* stabilize phyllotactic patterning. Genes & development.

[24] Benková E (2003). Local, efflux-dependent auxin gradients as a common module for plant organ formation. Cell.

[25] Paponov IA, Teale WD, Trebar M, Blilou I, Palme K (2005). The PIN auxin efflux facilitators: evolutionary and functional perspectives. Trends in plant science.

[26] Bayer EM (2009). *Integration of transpo*rt-based models for phyllotaxis and midvein formation. Genes & development.

[27] Heisler MG (2005). Patterns of auxin transport and gene expression during primordium development revealed by live imaging of the Arabidopsis inflorescence meristem. Current biology.

[28] Gallavotti A, Yang Y, Schmidt RJ, Jackson D (2008). The relationship between auxin transport and maize branching. Plant Physiology.

[29] Vieten A (2005). Functional redundancy of PIN proteins is accompanied by auxin-dependent cross-regulation of PIN expression. Development.

[30] Habets ME, Offringa R (2014). PIN‐driven polar auxin transport in plant developmental plasticity: a key target for environmental and endogenous signals. New Phytologist.

[31] Wenzel CL, Schuetz M, Yu Q, Mattsson J (2007). Dynamics of MONOPTEROS and PIN‐FORMED1 expression during leaf vein pattern formation in Arabidopsis thaliana. The Plant Journal.

[32] Hamann T, Benkova E, Bäurle I, Kientz M, Jürgens G (2002). The Arabidopsis BODENLOS gene encodes an auxin response protein inhibiting MONOPTEROS-mediated embryo patterning. Genes & Development.

[33] Garrett JJ (2012). A novel, semi-dominant allele of MONOPTEROS provides insight into leaf initiation and vein pattern formation. Planta.

[34] Růžička K (2007). Ethylene regulates root growth through effects on auxin biosynthesis and transport-dependent auxin distribution. The Plant Cell.

[35] Lewis DR, Negi S, Sukumar P, Muday GK (2011). Ethylene inhibits lateral root development, increases IAA transport and expression of PIN3 and PIN7 auxin efflux carriers. Development.

[36] Geldner N (2003). The Arabidopsis GNOM ARF-GEF mediates endosomal recycling, auxin transport, and auxin-dependent plant growth. Cell.

[37] Dhonukshe P (2008). Generation of cell polarity in plants links endocytosis, auxin distribution and cell fate decisions. Nature.

[38] Dhonukshe P (2010). Plasma membrane-bound AGC3 kinases phosphorylate PIN auxin carriers at TPRXS (N/S) motifs to direct apical PIN recycling. Development.

[39] Friml J (2004). A PINOID-dependent binary switch in apical-basal PIN polar targeting directs auxin efflux. Science.

[40] Michniewicz M (2007). Antagonistic regulation of PIN phosphorylation by PP2A and PINOID directs auxin flux. Cell.

[41] Zourelidou M (2009). The polarly localized D6 PROTEIN KINASE is required for efficient auxin transport in Arabidopsis thaliana. Development.

[42] Abas L (2006). Intracellular trafficking and proteolysis of the Arabidopsis auxin-efflux facilitator PIN2 are involved in root gravitropism. Nature cell biology.

[43] Laxmi A, Pan J, Morsy M, Chen R (2008). Light plays an essential role in intracellular distribution of auxin efflux carrier PIN2 in Arabidopsis thaliana. PloS one.

[44] Santner AA, Watson JC (2006). The WAG1 and WAG2 protein kinases negatively regulate root waving in Arabidopsis. The Plant Journal.

[45] Huang F (2010). Phosphorylation of conserved PIN motifs directs Arabidopsis PIN1 polarity and auxin transport. The Plant Cell.

[46] Okada K, Ueda J, Komaki MK, Bell CJ, Shimura Y (1991). Requirement of the auxin polar transport system in early stages of Arabidopsis floral bud formation. The Plant Cell.

[47] Guenot B (2012). PIN1-independent leaf initiation in Arabidopsis. Plant Physiology.

[48] Koenig D, Bayer E, Kang J, Kuhlemeier C, Sinha N (2009). Auxin patterns Solanum lycopersicum leaf morphogenesis. Development.

[49] Zhang X-l, Qi M-f, Xu T, Lu X-j, Li T-l (2015). Proteomics profiling of ethylene-induced tomato flower pedicel abscission. Journal of proteomics.

[50] Zhang J, Nodzyński T, Pěnčík A, Rolčík J, Friml J (2010). PIN phosphorylation is sufficient to mediate PIN polarity and direct auxin transport. Proceedings of the National Academy of Sciences.

[51] Lv D-W (2014). *Proteome and phosphoproteome chara*cterization reveals new response and defense mechanisms of Brachypodium distachyon leaves under salt stress. Molecular & Cellular Proteomics.

[52] Ulmasov T, Murfett J, Hagen G, Guilfoyle TJ (1997). Aux/IAA proteins repress expression of reporter genes containing natural and highly active synthetic auxin response elements. The Plant Cell.

[53] Bai F, DeMason DA (2006). Hormone interactions and regulation of Unifoliata, PsPK2, PsPIN1 and LE gene expression in pea (Pisum sativum) shoot tips. Plant and cell physiology.

[54] Sieberer T (2000). Post-transcriptional control of the Arabidopsis auxin efflux carrier EIR1 requires AXR1. Current Biology.

[55] Abeles F, Rubinstein B (1964). Regulation of ethylene evolution and leaf abscission by auxin. Plant Physiology.

[56] Rubinstein B, Leopold A (1963). Analysis of the auxin control of bean leaf abscission. Plant physiology.

[57] Meir S, Hunter DA, Chen J-C, Halaly V, Reid MS (2006). Molecular changes occurring during acquisition of abscission competence following auxin depletion in Mirabilis jalapa. Plant Physiology.

[58] Meir S, Hunter D, Jen-Chih C, Reid M (2003). Molecular study of the acquisition of increased sensitivity to ethylene in the abscission zone in response to removal of the auxin source. NATO SCIENCE SERIES SUB SERIES I LIFE AND BEHAVIOURAL SCIENCES.

[59] Wang S, Tiwari SB, Hagen G, Guilfoyle TJ (2005). AUXIN RESPONSE FACTOR7 restores the expression of auxin-responsive genes in mutant Arabidopsis leaf mesophyll protoplasts. The Plant Cell.

[60] Jain RN, Brunkan CS, Chew CS, Samuelson LC (2006). Gene expression profiling of gastrin target genes in parietal cells. Physiological genomics.

[61] Ma C (2015). A KNOTTED1-LIKE HOMEOBOX protein regulates abscission in tomato by modulating the auxin pathway. Plant physiology.

[62] Livak KJ, Schmittgen TD (2001). Analysis of relative gene expression data using real-time quantitative PCR and the 2− ΔΔCT method. methods.

[63] Pirttilä AM, Laukkanen H, Pospiech H, Myllylä R, Hohtola A (2000). Detection of intracellular bacteria in the buds of Scotch pine (Pinus sylvestris L.) by *in situ* hybridization. Applied and Environmental Microbiology.

[64] Liu Y, Schiff M, Dinesh‐Kumar S (2002). Virus‐induced gene silencing in tomato. The Plant Journal.

[65] Sakamoto K, Briggs WR (2002). Cellular and subcellular localization of phototropin 1. The Plant Cell.

[66] Yang J, Zhang J, Wang Z, Zhu Q, Wang W (2001). Hormonal changes in the grains of rice subjected to water stress during grain filling. Plant Physiology.

[67] He, Z. A laboratory guide to chemical control technology on field crop. *Beijing Agr*. *Univ*. *Press, Beijing, China* (1993).

[68] Sparkes IA, Runions J, Kearns A, Hawes C (2006). Rapid, transient expression of fluorescent fusion proteins in tobacco plants and generation of stably transformed plants. Nature protocols.

