# Supplementary material for: SlPIN1 regulates auxin efflux to affect flower abscission process
Source: Sci Rep. 2017 Nov 2;7:14919. doi: 10.1038/s41598-017-15072-7 (PMC5668252; doi:10.1038/s41598-017-15072-7)
Supplement: Supplementary file 1 — Supplementary Information [file 41598_2017_15072_MOESM1_ESM.pdf]

SlPIN1 regulates auxin efflux to affect flower abscission process

Zihang Shi[1,2*], Yun Jiang[1,2*], Xinqi Han[1,2], Xin Liu[1,2], Ruishu Cao[1,2], Mingfang Qi[1,2], Tao Xu[1,2]& Tianlai Li[1,2]

1 College of Horticulture, Shenyang Agricultural University, Shenyang 110866, Liaoning, People' s Republic of China. 2 Key Laboratory of Protected Horticulture of Ministry of Education, No. 120 Dongling Road, Shenhe District 110866, People' s Republic of China. *These authors contributed equally to this work. Correspondence and requests for materials should be addressed to T.X. (email: syauxutao@qq.com) and T.L. (email: tianlaili@126.com)

Supplementary information

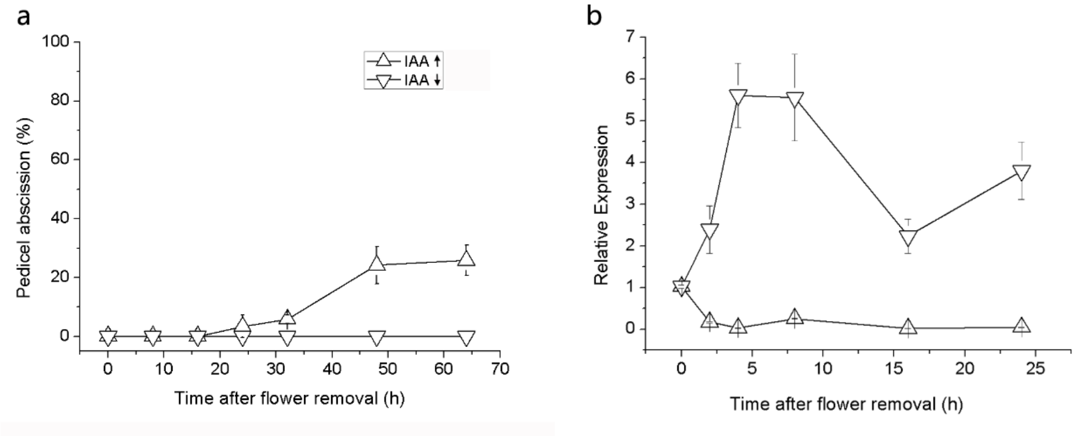

Supplementary Figure S1. Abscission process and *SlPIN1* expression profiles following the IAA treatments at different ends. The arrows indicate the opposite directions of incubation. The data are presented as the mean (± SD) values corresponding to three independent tests. For each test of abscission rate, 40 to 50 pedicels were investigated.

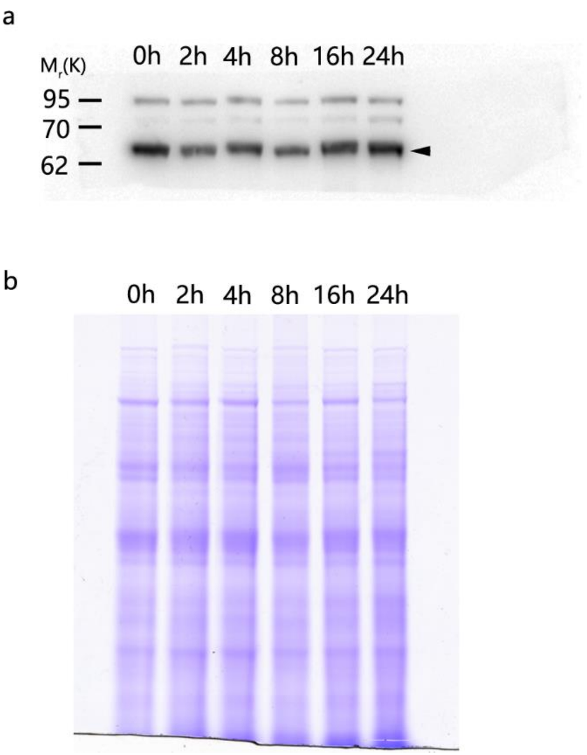

Supplementary Figure S2. Full-length images in SlPIN1 western blotting experiments. a, SlPIN1 western blotting of the AZ protein samples as described in Fig. 3 a. A specific signal among the 62-70 KD (arrowhead) was detected. b, the Coomassie-staining of the AZ protein samples.

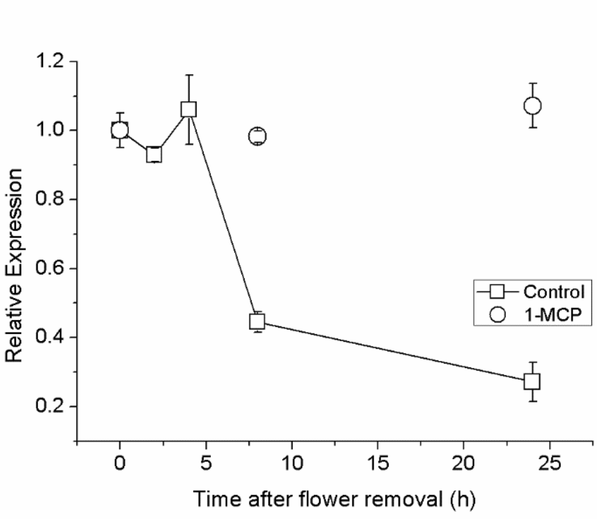

Supplementary Figure S3. ***FEI2*** **expression profiles in the AZ during abscission.** *FEI2* expression decreased at 8-24 h after flower removal, while 1-MCP treatment prevented the down-regulation of *FEI2* during abscission. The data are presented as the mean (±S D) values corresponding to three independent tests.

Supplementary Table S1. Primers used in this study

Primer name	Sequence (5'-3')
Forward primer of *SlPIN1* for RT-PCR	CAGGCAGCTCTACCACAAGG
Reverse primer of *SlPIN1* for RT-PCR	TGTAATCGGCAACGCAATC
Forward primer of *FEI2* for RT-PCR	TCTGGTCTCCACTGCTCAAGA
Reverse primer of *FEI2* for RT-PCR	CCAAGGGAACCGTAGCACAG
Forward primer for vector pSPT19 construction (Eco RI)	GG*GAATTC*ACTTATTGTTCTTGGGGTTCTTGCT
Reverse primer for vector pSPT19 construction (Hind III)	GG*AAGCTT*TTTTCCATCTTCTTTTAACTCTGCT
Forward primer of *SlPIN1* for VIGS construction (Eco RI)	CCG*GAATTC*TGGTACTGCTGGGAGAAACTCAAAC
Reverse primer of *SlPIN1* for VIGS construction (BamHI)	CGC*GGATCC*GCTTTGTTGCCATTGTTACTAGGTG
Forward primer of *SlPIN1* for bait construction of Y2H (Eco RI)	CCG*GAATTC*AGAGGTGCAAGACTGCTAATTTCT
Reverse primer of *SlPIN1* for bait construction of Y2H (BamHI)	CGC*GGATCC*GGACTGTGCAATTATAGCAGGC
Forward primer of *SlPIN1* for vector construction of BiFC (KpnI)	CGG*GGTACC*AGAGGTGCAAGACTGCTAATTTCT
Reverse primer of *SlPIN1* for vector construction of BiFC (SpeI)	CGG*ACTAGT*GGACTGTGCAATTATAGCAGGC
Forward primer of *PP2C* for vector construction of BiFC (KpnI)	CGG*GGTACC*ATGACTGGAGGCAAAGAAATCATCG
Reverse primer of *PP2C* for vector construction of BiFC (Eco RI)	CCG*GAATTC*TTGGAACCTTACAACTACGCAGG

Supplementary Table S2. Phosphopeptides of SlPIN1

Phosphorylated peptide and site (in lowercase)	pRS Score	Mascot Score	Relative quantity	
			12h/0h	24h/0h
DDFsFANR	108	35	0.582	0.378
FNNYHGGAAAPQsNsNtHyPAPNPGMFsPsNNGNk	57	11	0.700	0.761
